# Is the METS-IR Index a Potential New Biomarker for Kidney Stone Development?

**DOI:** 10.3389/fendo.2022.914812

**Published:** 2022-07-14

**Authors:** Xudong Shen, Yang Chen, Yan Chen, Hu Liang, Guoxiang Li, Zongyao Hao

**Affiliations:** ^1^ Department of Urology, the First Affiliated Hospital of Anhui Medical University, Hefei City, China; ^2^ Institute of Urology, Anhui Medical University, Hefei City, China; ^3^ Anhui Province Key Laboratory of Genitourinary Diseases, Anhui Medical University, Hefei City, China; ^4^ Department of General Practice, Wuhu City Second People`s Hospital, Wuhu City, China

**Keywords:** metabolic syndrome, kidney stones, METS-IR index, insulin resistance, NHENSE (National Health and Nutrition Examination Survey)

## Abstract

**Objective:**

The purpose of this study was to examine whether the METS-IR index is associated with kidney stones in American adults.

**Method:**

Participants from the National Health and Nutrition Examination Survey (NHANES) database from 2007-2018 were selected for logistic regression analysis, subgroup analyses, and the calculation of dose-response curves to assess the association between the METS-IR index and the incidence of kidney stones.

**Result:**

This study enrolled 30,612 adults aged >20 years, 2901 of whom self-reported having had kidney stones in the past. And, after controlling for potential confounders, each unit increase in the METS-IR index was linked with a 1.23 percent rise in kidney stone incidence (OR= 1.0123, 95% CI: 1.0092 - 1.0155), with stratified analysis indicating that this was true in all subgroups. Between all groups, an elevated METS-IR index was related to kidney stone formation, and the dose-response curve revealed a positive non-linear connection between METS-IR index and kidney stone risk, with a threshold effect analysis revealing an inflection point value of 50.8314.

**Conclusion:**

Higher METS-IR index is associated with the occurrence of kidney stones,and while no causative association can be shown, this is cause for concern.

## Introduction

The kidney stone is a benign disease that affects the renal calyces, renal pelvis, and the junction of the renal pelvis and ureter and is one of the most common in urology (1). Current prevalence levels of kidney stones are high, and they have been increasing globally throughout the past few decades (2). According to the most recent survey study of the National Health and Nutrition Examination Survey (3), the prevalence of kidney stones is as high as 11% in the United States, 9% in Europe (1),and 5.8% in China (4). Presently, minimally invasive endoscopic procedures such as percutaneous nephrolithotomy, flexible ureteroscopic lithotripsy and other endoscopic procedures are routinely used to treat kidney stones.A high recurrence risk exists even after completion of treatment (5). If not effectively treated, it may result in serious complications such as irreversible kidney damage and end-stage renal disease.It is becoming increasingly apparent that kidney stones are a significant public health concern, as well as a major economic burden for the healthcare system (6).

As a result of the high rates of recurrence and incidence of kidney stones, prevention should be considered a high priority.A multitude of systemic factors have been reported to be associated with an increased risk of kidney stones, suggesting that genetic, environmental, and nutritional influences may play an important role in stone development (7). It has become increasingly apparent that modifiable factors, including diet and lifestyle, can influence kidney stone development. Every time the conditions of living improve, the number of people suffering from metabolic syndrome increases due to high-fat and high-sugar diets. Metabolic syndrome (MetS) refers to a group of metabolic disorders, which include obesity (primarily abdominal obesity), fasting, postprandial hyperglycemia, hypertension, and dyslipidemia. It has been suggested that these conditions may be linked to a common mechanism: insulin resistance(IR) (8, 9). Increasingly, studies confirm an association between IR and major kidney stones. It is believed that IR increases the risk of urinary calcium stones by reducing the excretion of citrate in the urine (10). Metabolic syndrome components may contribute to the development of kidney stones through subclinical hyperinsulinemia and insulin resistance (11).

Hyperinsulinemic normoglycemic clamps (HECs) are currently the gold standard for assessing insulin sensitivity in peripheral tissues (12). Due to the complexity, time consuming nature, and resource consuming nature of this method, simpler metrics are often used to assess insulin resistance. The METS-IR index, a new metric for measuring insulin resistance (IR) as a simple, reliable, and reproducible predictive metric, has been proposed in 2018 (12, 13). Given the METS-IR index’s role as a marker for IR, a possible correlation between the METS-IR index and renal calculi might be posited. Nevertheless, no previous study has investigated the relationship between the METS-IR index and kidney stones. Therefore, in the present study, we aimed to assess the value of the METS-IR index in the incidence of kidney stones in the United States (US) population.

## Materials and Methods

### Study Population

**Figure 1:**
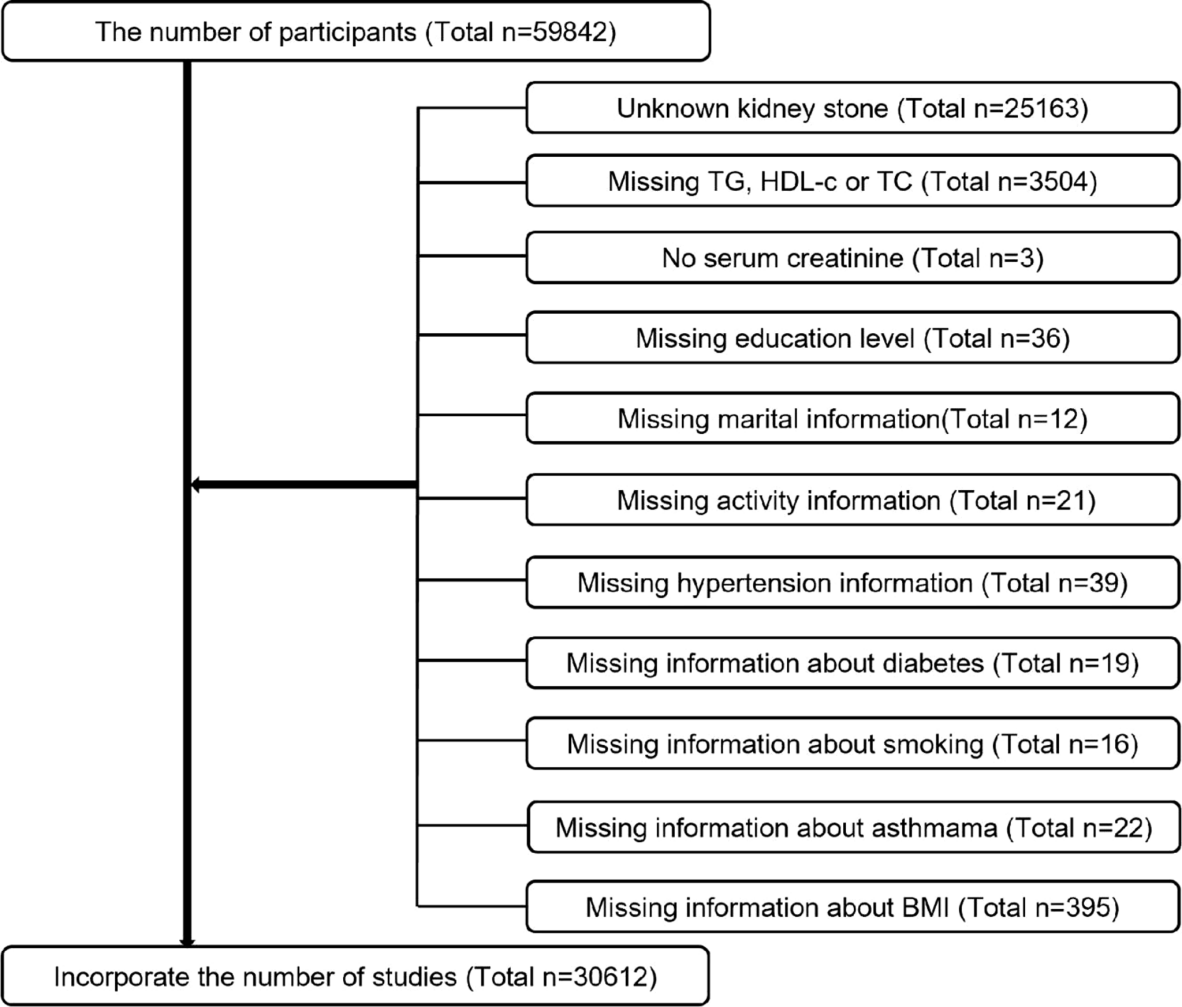
Sample selection flowchart from NHANES 2007–2018.

Data for this study were obtained from the NHANES database based on big data mining methods and were conducted by the Centers for Disease Control and Prevention (CDC) (14, 15). NCHS’s Institutional Review Board reviewed and approved the study protocol, as well as forms of consent signed by participants. Six consecutive two-year survey cycles, including the Kidney Stone Questionnaire, were used for the evaluation of our research. All participants were assessed with the KIQ026 survey (Do You Have Kidney Stones) and 59,842 completed the questionnaire. Exclusion criteria were as follows (**Figure 1**). Ultimately, 30612 cases were included in the study, including 2901 patients who self-reported renal calculi.

### Data Collection and Definition

METS-IR index is intended to be used as an exposure variable. METS-IR= Ln((2 × fasting glucose) + fasting triglycerides) × body mass index)/[Ln(high-density lipoprotein cholesterol)]. An automated biochemical analyzer was used to determine fasting glucose and triglyceride concentrations. The serum triglyceride concentrations were measured using a Roche Modular P chemistry analyzer and a Roche Cobas 6000 chemistry analyzer. In addition, the questionnaire KIQ026 consists of a question to determine whether the respondent has ever experienced kidney stones. Studies have verified the validity of self-reported kidney stone status (3). A participant was deemed to have kidney stones if he answered “yes” to the question of whether he had ever experienced kidney stones. The outcome variable for this research was the occurrence of kidney stones.

An adjusted multivariate model was used to summarize potential confounders that might confound the association between METS-IR index and kidney stones. Covariates in our study included sex (male/female), age (years), race, education level, poverty to income ratio (PIR), marital status (married or living with partner/single), alcohol consumption (drinking or not), physical activity (vigorous/moderate/below moderate), cholesterol level (mg/dl), serum creatinine (mg/dl), blood calcium (mg/dl), albumin creatinine ratio (mg/g), body mass index (BMI), smoking status (smoking or not), hypertension, diabetes, asthma [a proven risk factor for stones (16)] and dietary intake factors including energy intake, fat intake, sugar intake and water intake, all participants were eligible for two 24-hour dietary recalls and our the average consumption of the two recalls will be used in our analysis. When missing values were less than 10% (17, 18), median values were directly used as a proxy. However, when missing values exceeded 10%, we converted these values into categorical variables and assessed them in tertiles, with the lowest tertile serving as a reference. On the CDC’s website at www.cdc.gov/nchs/nhanes, you can find detailed information on all the measurement procedures used in the study.

### Statistical Methods

A suitable NHANES sample weight was employed and the complicated multistage cluster survey design was accounted for in the analysis. Variables with continuous characteristics were expressed as means together with their standard deviations, and categorical characteristics were expressed as percentages. In order to determine the variability of clinical characteristics among groups, weighted Chi-square tests (categorical variables) and weighted variance analysis (continuous variables with a normally distributed distribution) or weighted Kruskal-Wallis`s H tests (continuous variables with a skewed distribution) were employed. Based on guidelines (19), multiple logistic regression models were used to examine the independent relationship between METS-IR index and different tertile groups of METS-IR index and kidney stones. In model 1, no adjustment for covariates was made. Model 2 was adjusted for sex, age, and race. Model 3 was adjusted for sex, age, race, education level, poverty-income ratio, marital status, alcohol intake, physical activity, cholesterol, serum creatinine, smoking status, hypertension, diabetes, asthma, energy intake, fat intake, sugar intake, and water intake. For further assessment of the relationship between METS-IR index and kidney stones, smooth curve fitting (penalized spline method) and generalized additive model (GAM) regression were conducted. Additionally, univariate linear regression models and two-piecewise linear regression models were constructed using the same covariates. To identify the best model, it was also necessary to conduct a logarithmic ratio test. The model was additionally used to determine whether a threshold exists. The inflection point connecting the segments based on the model had the highest likelihood, and was determined using a two-step recursive methodology. Moreover, an interaction term was added using a log-likelihood ratio test model in order to examine the heterogeneity of the association between subgroups. A statistically significant value was considered to be p < 0.05.Empower^®^ software (www.empowerstats.com; X&Y Solutions, Inc., Boston, MA, USA) and R 3.4.3 (http://www.r-project.org, The R Foundation) were used to conduct all analyses.

## Results

An overview of the baseline demographic characteristics of the participants is provided in **Table 1**. There was a significant difference in the METS-IR index between the stone and non-stone groups, 47.291 ± 13.591vs 43.259 ± 12.936, p< 0.001.

**Table 1:** Baseline characteristics of participants,weighted.

Characteristic	Nonstone formers	Stone formers	P-value
	N=27711	N=2901	
Age (years)	46.812 ± 16.806	53.299 ± 15.576	<0.001
PIR	2.965 ± 1.600	2.982 ± 1.572	0.56
BMI (kg/m^2^)	28.927 ± 6.829	30.623 ± 6.992	<0.001
Serum Cholesterol (mg/dl)	194.041 ± 41.436	192.268 ± 42.519	0.026
Serum Calcium (mg/dl)	9.392 ± 0.358	9.370 ± 0.379	0.016
Serum Creatinine (mg/dl)	0.875 ± 0.328	0.937 ± 0.570	<0.001
Urine Albumin Creatinine Ratio (mg/g)	31.026 ± 265.850	42.047 ± 267.225	<0.001
METS-IR Index	43.259 ± 12.936	47.291 ± 13.591	<0.001
Gender (%)			<0.001
Male	47.355	54.689	
Female	52.645	45.311	
Race (%)			<0.001
Mexican American	14.772	11.198	
White	65.680	76.974	
Black	11.301	5.717	
Other Race	8.247	6.112	
Education Level (%)			0.005
Less than high school	20.372	19.862	
High school	28.532	31.305	
More than high school	51.097	48.833	
Marital Status (%)			<0.001
Cohabitation	63.286	69.099	
Solitude	36.714	30.901	
Alcohol (%)			0.486
Yes	60.328	59.463	
No	18.519	19.386	
Unclear	21.152	21.151	
High Blood Pressure (%)			<0.001
Yes	30.098	46.704	
No	69.902	53.296	
Diabetes (%)			<0.001
Yes	8.752	17.998	
No	91.248	82.002	
Smoked			<0.001
Yes	43.575	49.511	
No	56.425	50.489	
Physical Activity (%)			<0.001
Never	26.738	31.021	
Moderate	31.829	31.227	
Vigorous	41.433	37.753	
Asthma (%)			<0.001
No	85.474	82.656	
Yes	14.526	17.344	
Total Kcal (%)			0.045
Tertile 1	24.748	24.100	
Tertile 2	28.386	30.899	
Tertile 3	30.846	30.930	
Unclear	16.020	14.071	
Total Sugar (%)			0.174
Tertile 1	23.723	24.460	
Tertile 2	24.612	23.062	
Tertile 3	24.438	25.611	
Unclear	27.226	26.867	
Total Water (%)			0.005
Tertile 1	23.778	22.598	
Tertile 2	28.903	30.070	
Tertile 3	31.299	33.261	
Unclear	16.020	14.071	
Total Fat (%)			0.005
Tertile 1	23.778	22.598	
Tertile 2	28.903	30.070	
Tertile 3	31.299	33.261	
Unclear	16.020	14.071	

Statistically significant: p<0.05; Mean+SD for continuous variables: P value was calculated by weighted linear regression model.

%for Categorical variables: P value was calculated by weighted chi-square test.

BMI, Body mass index (kg/m^2^); PIR, Ratio of family income to poverty.

### Increased METS-IR Index Is Associated With a Higher Risk of Kidney Stones

The METS-IR index was positively related to the presence of kidney stones. In the fully adjusted model (model 3), the positive association remained stable (OR=1.0126, 95% CI: 1.0095 - 1.0158), indicating that a unit increase in METS-IR index was associated with a 1.26 percent increase in the risk of kidney stones. Additionally, we converted the METS-IR index from a continuous variable into a categorical variable (tertile) prior to performing a sensitivity analysis. In Tertile 2 and Tertile 3, the likelihood of kidney stones occurrence increased by 36.11% and 59.42%, respectively, compared with the lowest METS-IR index in the lowest tertile (Tertile 1), as illustrated in **Table 2**.

**Table 2:** Analysis between METS-IR index with kidney stone formation.

Characteristic	Model 1 OR (95%CI)	Model 2 OR (95%CI)	Model 3 OR (95%CI)
METS-IR Index	1.0189 (1.0161, 1.0216)	1.0192 (1.0163, 1.0221)	1.0123 (1.0092, 1.0155)
Categories			
Tertile 1	1	1	1
Tertile 2	1.6182 (1.4602, 1.7933)	1.4631 (1.3175, 1.6249)	1.3611 (1.2239, 1.5137)
Tertile 3	2.0475 (1.8540, 2.2610)	1.9429 (1.7552, 2.1508)	1.5942 (1.4312, 1.7757)

Model 1 = no covariates were adjusted.

Model 2 = Model 1+ age,gender, race were adjusted.

Model 3 = Model 2+ gender, diabetes, blood pressure, education, marital status, serum calcium, PIR, asthma, total water, total kcal, total fat, total sugar, smoked, physical activity, alcohol use, serum creatinine, serum cholesterol, urine albumin creatinine ratio were adjusted.

### Metrics-IR’s Dose Response and Threshold Effect on Kidney Stones

**Figure 2:**
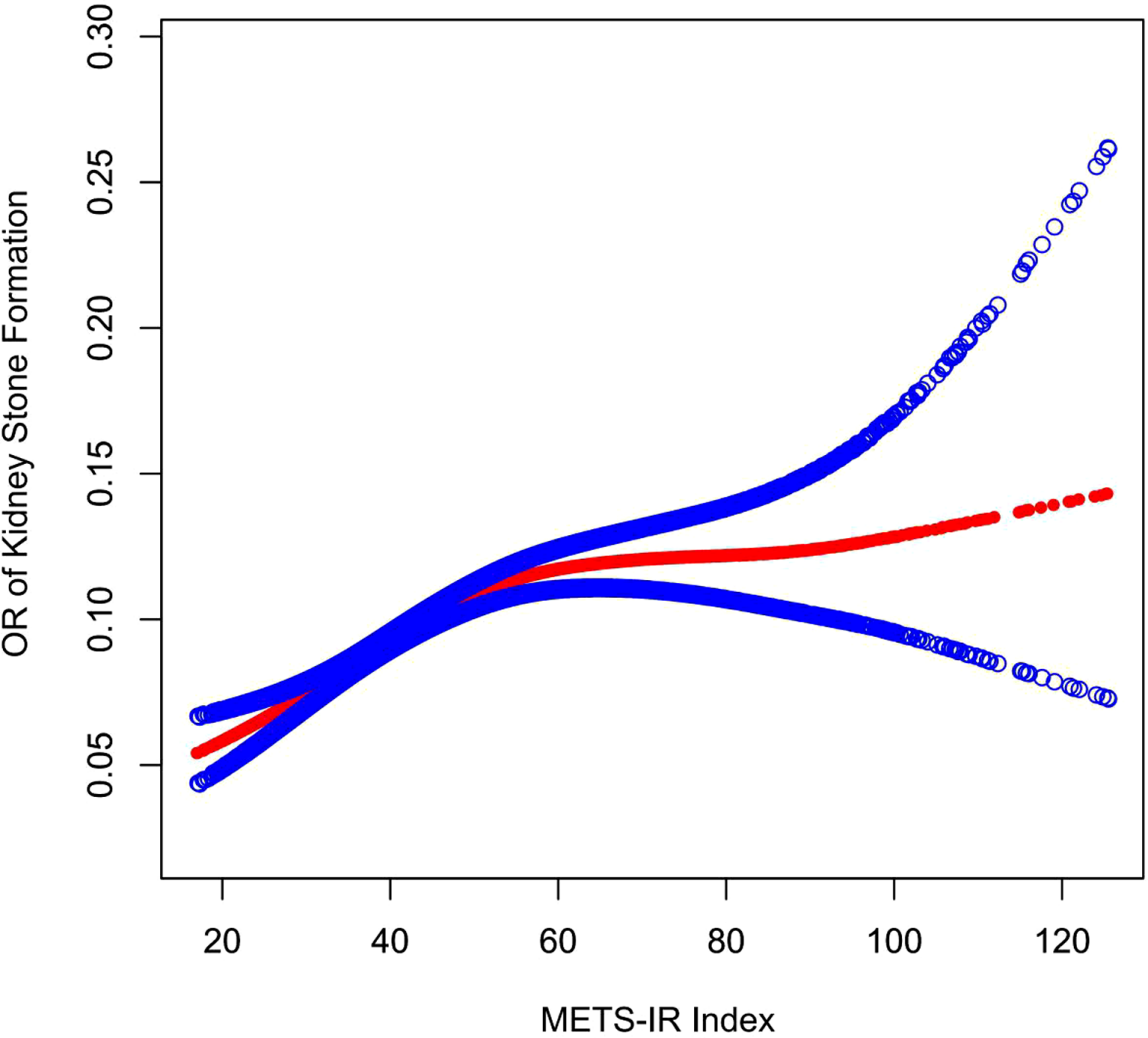
Density dose–response relationship between METS-IR index with kidney stone formation. The area between two blue dotted lined is expressed as a 95% CI. Each point shows the magnitude of the METS-IR index and is connected to form a continuous line. Adjusted for all covariates except effect modifier.

A generalized additive model and smoothed curve fitting have been used to analyze the relationship between METS-IR index and kidney stones. Results of our study demonstrated a nonlinear relationship between METS-IR index and kidney stones (**Figure 2** and **Table 3**). Based on a two-segment linear regression model, the METS-IR inflection point was calculated at 50.8314.As shown in **Table 3**, the OR on the left side of the inflection point was 1.0238 (95% CI: 1.0178-1.0299), whereas the OR on the right side of the inflection point was 1.0015 (log-likelihood ratio test, p < 0.001).

**Table 3:** Two-piecewise linear regression and logarithmic likelihood ratio test explained the threshold effect analysis of METS-IR index on kidney stone.

METS-IR Index	ULR Test	PLR Test	LRT test
	OR (95% CI)	OR (95% CI)	P value
<50.8314umol/L	1.0121 (1.0089, 1.0152)	1.0238 (1.0178, 1.0299)	<0.0001
≥50.8314umol/L		1.0015 (0.9957, 1.0073)	

ULR, univariate linear regression; PLR, piecewise linear regression; LRT, logarithmic likelihood ratio test, statistically significant: p < 0.05.

### Subgroup Analysis

Subgroup analyses were performed in order to assess the robustness of the association between METS-IR index and kidney stones. All subgroup analyses indicated that an increased METS-IR index was positively associated with kidney stone occurrence (**Table 4**). We also tested for interactions between age, gender, hypertension, and diabetes mellitus.

**Table 4:** Subgroup analysis between METS-IR index with kidney stone formation.

Characteristic	Model 1 OR (95%CI)	Model 2 OR (95%CI)	Model 3 OR (95%CI)	p for trend*	p for interaction*
Stratified by gender					0.4694
Male	1.0179 (1.0140, 1.0218)	1.0185 (1.0144, 1.0227)	1.0123 (1.0078, 1.0168)	<0.001	
Female	1.0191 (1.0152, 1.0230)	1.0201 (1.0161, 1.0241)	1.0117 (1.0072, 1.0161)	<0.001	
Stratified by age (years)					0.7463
20-39	1.0157 (1.0101, 1.0214)	1.0158 (1.0101, 1.0215)	1.0068 (1.0004, 1.0132)	0.025	
40-59	1.0202 (1.0156, 1.0248)	1.0207 (1.0160, 1.0254)	1.0128 (1.0076, 1.0180)	<0.001	
60-80	1.0195 (1.0148, 1.0243)	1.0187 (1.0138,	1.0117 (1.0064, 1.0171)	<0.001	
Stratified by hypertension					0.0651
NO	1.0189 (1.0149, 1.0230)	1.0193 (1.0150, 1.0236)	1.0152 (1.0107, 1.0197)	<0.001	
YES	1.0102 (1.0063, 1.0142)	1.0122 (1.0080, 1.0164)	1.0074 (1.0029, 1.0118)	0.001	
Stratified by diabetes					0.3559
NO	1.0163 (1.0130, 1.0195)	1.0172 (1.0138, 1.0207)	1.0134 (1.0098, 1.0170)	<0.001	
YES	1.0094 (1.0036, 1.0152)	1.0108 (1.0045, 1.0170) 0.000670	1.0075 (1.0011, 1.0140)	<0.001	

Model 1 = no covariates were adjusted.

Model 2 = Model 1+age,gender,race were adjusted.

Model 3 = adjusted for all covariates except effect modifier.

*Means only in model 3.

## Discussion

Generally, kidney stones are a recurrent disease over the course of a lifetime. Recurrent stones are more likely to recur in the future and have a poorer prognosis (7).A low urine output, a high urinary calcium level, a high urinary uric acid level, a high oxaluria level, and an abnormal urinary pH level can all contribute to stone formation (20).Due to the complex etiology of stones, large individual differences, regional differences, and high recurrence rates, a comprehensive study of risk factors for stones and the search for factors associated with stone recurrence are necessary to guide treatment and prevention.

As far as our knowledge is concerned, this is the first study to investigate the relationship between the METS-IR index and kidney stones and to demonstrate the predictive significance of METS-IR in the development of kidney stones. The results of this large cross-sectional study reveal that higher METS-IR scores are positively associated with an increased risk of kidney stone formation. Individuals in the highest tertile of METS-IR had a 0.59-fold greater risk of developing new kidney stones as compared to those in the lowest tertile. Moreover, this study not only evaluated the independent effects of METS-IR and the risk of kidney stone development, but also examined the dose–response relationship between the two factors and derived a threshold effect for METS-IR of 59.8314.In comparison to the left side of the inflection point, when METS-IR was at 59.8314, there was an increasing trend in kidney stone occurrence with increasing METS-IR (OR =1.0238, 95% CI: 1.0178-1.0299); however, when METS-IR was at 59.8314, the trend gradually plateaued compared to the right side of the inflection point (OR =1.0015, 95% CI. 0.9957-1.0073).The factors included in this study were stratified by sex, age, hypertension, and diabetes status, while the interaction test p-values were not statistically significant, indicating that this association was independent of age, hypertension, and diabetes, suggesting that it could be applied to all types of populations. Intriguingly, our results suggest that an elevated METS-IR index is associated with a greater risk of kidney stone formation among non-hypertensive and non-diabetic individuals than among hypertensive and diabetic individuals. First, the possibility exists that those with diabetes and hypertension may be more cognizant of healthy eating, after all, a high sugar diet is more likely to contribute to the development of metabolic syndrome (21, 22). In addition, Iran found that non -hypertensive people`s IR resistance will cause cardiovascular disease, and there is no such effect in people with hypertension (23). Another Japanese study found that the increase in IR levels in non -diabetic people will lead to increasing coronary heart disease and stroke probability (24). Hedblad et al. (25) reported that the HOMAIR distribution of non-diabetic individuals has the 75th percentage value (2.12 for men, 1.80 (for women). The risk of infarction is significantly higher than those without these HOMA-IR values.These results suggest that IR resistance is more likely to cause more severe consequences in non-diabetic and non-hypertensive populations. Although the research objects are different, this result also confirms to some extent that our results may be correct. Considering these findings, it is also suggested that the association between METS-IR index and kidney stones should be more closely monitored among healthy people.

The METS-IR index was first reported in 2018 and is considered to be an intuitive and reliable measure of inflammation that can be used in clinical decision making (12, 13). In a cohort study conducted in Korea, Kim et al. demonstrated that IR was associated with the development of kidney stones in Korean men (26). Another study from Japan also confirmed that metabolic syndrome can cause insulin resistance, which in turn increases the risk of kidney stones (11). There has been some research on the influence of IR on kidney stone formation as well. Insulin resistance has been shown to cause ammonia production to decline and sodium and bicarbonate reabsorption to increase, which results in a decrease in the pH of the urine (27). Likewise, insulin resistance may result in a reduction in renal ammonia production, which will lead to a reduction in ammonia buffering and further decrease urine pH (28). In urine with a pH below 5.5, less soluble uric acid is formed from urate, which can cause uric acid stones (29). Further, IR increases renal tubule citrate uptake and decreases urinary citrate levels (10), which is one of the primary causes of calcium stones. In light of the fact that METS-IR index and IR levels are positively correlated, it may be possible to explain why a higher METS -IR index is related to an increased risk of kidney stones.

Several advantages are associated with our study. The NHANES is a representative sample of the US population. It strictly adheres to a well-designed study protocol with high standards of quality assurance and quality control. Moreover, our results are robust when tested against a range of sensitivity analyses that confirm our primary analysis. Nonetheless, we recognize the limitations of our study. As a result of using the NHANES database, a cross-sectional study, we were not able to investigate a causal relationship between METS-IR and kidney stones. Additionally, the diagnosis of kidney stones was made on the basis of a questionnaire, which was unable to provide information on the size and type of kidney stones, and was susceptible to recall bias; and finally, detailed clinical variables, such as medication history and kidney stone type, were not included in the database and required further investigation. Upon confirmation of our findings, an RCT study in a multicenter environment will be conducted. In spite of these limitations, this study has the strength of suggesting a new index of kidney stone incidence and demonstrating the relationship between METS-IR scores and kidney stones.

## Summary

According to the results of this cross-sectional analysis of a representative sample, a high METS-IR index is associated with an increase in the prevalence of kidney stones. Mets-IR shows promise as a new marker that can help guide prevention of kidney stones

## Data Availability Statement

The datasets presented in this study can be found in online repositories. The names of the repository/repositories and accession number(s) can be found in the article/supplementary material.

## Ethics Statement

The studies involving human participants were reviewed and approved by The NCHS Research Ethics Review Committee approved the NHANES survey protocol (https://www.cdc.gov/nchs/nhanes/irba98.htm). The patients/participants provided their written informed consent to participate in this study.

## Author Contributions

XS, YangC, YanC, HL, and GL performed the material preparation, collected the data, and analyzed the data. ZH wrote the original draft of the manuscript. All authors contributed to the study conception and design and commented on the previous versions of the manuscript, and read and approved the final manuscript.

## Funding

This work was supported by the National Natural Science Foundation of China (82070724), Natural Science Foundation of Anhui Province(1908085MH246).

## Conflict of Interest

The authors declare that the research was conducted in the absence of any commercial or financial relationships that could be construed as a potential conflict of interest.

## Publisher’s Note

All claims expressed in this article are solely those of the authors and do not necessarily represent those of their affiliated organizations, or those of the publisher, the editors and the reviewers. Any product that may be evaluated in this article, or claim that may be made by its manufacturer, is not guaranteed or endorsed by the publisher.

## Abbreviations

MetS, Metabolic syndrome; IR, Insulin resistance; OR, Odds ratio; BMI, Body mass index; US, United States; PIR, Ratio of family income to poverty.
